# Three Members of Polyamine Modulon under Oxidative Stress Conditions: Two Transcription Factors (SoxR and EmrR) and a Glutathione Synthetic Enzyme (GshA)

**DOI:** 10.1371/journal.pone.0124883

**Published:** 2015-04-21

**Authors:** Akihiko Sakamoto, Yusuke Terui, Taketo Yoshida, Taku Yamamoto, Hideyuki Suzuki, Kaneyoshi Yamamoto, Akira Ishihama, Kazuei Igarashi, Keiko Kashiwagi

**Affiliations:** 1 Faculty of Pharmacy, Chiba Institute of Science, Choshi, Chiba, Japan; 2 Graduate School of Science and Technology, Kyoto Institute of Technology, Kyoto, Kyoto, Japan; 3 Department of Frontier Bioscience, Hosei University, Koganei, Tokyo, Japan; 4 Graduate School of Pharmaceutical Sciences, Chiba University, Chiba, Chiba, Japan; 5 Amine Pharma Research Institute, Innovation Plaza at Chiba University, Chiba, Chiba, Japan; ENEA, ITALY

## Abstract

Members of polyamine modulon whose synthesis is enhanced at the level of translation were looked for under oxidative stress conditions caused by 0.6 μM K_2_TeO_3_. When an *Escherichia coli* polyamine-requiring mutant MA261 was cultured in the presence of K_2_TeO_3_, the degree of polyamine stimulation of cell growth was greater than in cells cultured in the absence of K_2_TeO_3_. Under these conditions, synthesis of SoxR, a transcriptional factor for expression of the superoxide response regulon, EmrR, a negative transcriptional factor for expression of the genes for drug excretion proteins, EmrA and EmrB, and of GshA, γ-glutamylcysteine synthetase necessary for glutathione (GSH) synthesis, were stimulated by polyamines at the level of translation. Polyamine stimulation of SoxR and EmrR synthesis was dependent on the existence of an unusually located Shine-Dalgarno (SD) sequence in *soxR* and *emrR* mRNAs. Polyamine stimulation of GshA synthesis was due to the existence of the inefficient initiation codon UUG instead of AUG. Polyamine stimulation of the synthesis of EmrR was mainly observed at the logarithmic phase of growth, while that of the synthesis of SoxR and GshA was at the stationary phase. These results strongly suggest that polyamines are involved in easing of oxidative stress through stimulation of synthesis of SoxR, EmrR and GshA together with RpoS, previously found as a member of polyamine modulon at the stationary phase.

## Introduction

Polyamines (putrescine, spermidine and spermine), aliphatic cations present in almost all living organisms, are necessary for normal cell growth [1]. Because polyamines interact with nucleic acids and exist mostly as polyamine-RNA complexes in cells [2, 3], their proliferative effects are presumed to be caused by changes in RNA function. In this context, it has been reported that polyamines stimulate the synthesis of some proteins *in vitro* [4, 5], increase the fidelity of protein synthesis [6, 7], and induce *in vivo* assembly of 30S ribosomal subunits [8, 9], suggesting that polyamines regulate protein synthesis at several different steps.

Previously we found that translation of a defined set of proteins in the polyamine-requiring mutant *Escherichia coli* MA261 is enhanced by polyamines [10]. We proposed that a set of genes whose expression is enhanced by polyamines at the level of translation can be classified as a “polyamine modulon” [10]. We have thus far identified 17 different genes as components of the polyamine modulon in *E*. *coli* [10–13]. There are several mechanisms underlying polyamine stimulation of the synthesis of various members of the polyamine modulon. First, polyamine stimulation of protein synthesis can occur when a Shine-Dalgarno (SD) sequence in mRNA is obscure or is distant from the initiation codon AUG. Polyamines cause structural changes of a region of the SD sequence and the initiation codon AUG, facilitating formation of the initiation complex. This is the case for *oppA*, *fecI*, *fis*, *rpoN*, *hns*, *rpoE*, *stpA*, *rmf*, *rpoZ* and *cpxR*. Second, polyamines enhance the inefficient initiation codon UUG- and GUG-dependent fMet-tRNA binding to ribosomes. This is the case for *cya*, *spoT*, *cra*, *uvrY* and *frr*. Third, polyamines stimulate read-through of the amber codon UAG-dependent Gln-tRNA^supE^ on ribosome-associated *rpoS* mRNA, or stimulate a +1 frameshift at the 26th UGA codon of *prfB* mRNA encoding RF2. The functions of 17 proteins encoded by polyamines were summarized [14], indicating that polyamines increase cell growth as well as cell viability.

In this study, we looked for new members of polyamine modulon under oxidative stress conditions. When *E*. *coli* cells were treated with K_2_TeO_3_, an inducer of oxidative stress [15, 16], the degree of polyamine stimulation of cell growth increased. Under these conditions, it was found that synthesis of two transcription factors (SoxR and EmrR) and γ-glutamylcysteine synthetase (GshA) catalyzing the first step of glutathione (GSH) synthesis [17–20] was enhanced by polyamines at the level of translation. Thus, roles of three proteins under oxidative stress conditions were evaluated.

## Materials and Methods

### Bacterial Strains and Culture Conditions

A polyamine-requiring mutant of *E*. *coli* MA261 (*speB speC gly leu thr thi*) [21] and MA261 *lacZ*::*Em* [22] were cultured in medium A [0.4% glucose (22.4 mM), 40.2 mM K_2_HPO_4_, 22.1 mM KH_2_PO_4_, 1.7 mM sodium citrate, 7.6 mM (NH_4_)_2_SO_4_, 0.41 mM MgSO_4_, 6 μM thiamine, 40 μM biotin, 0.8 mM leucine, 0.8 mM threonine, 0.7 mM methionine, 1 mM serine, 1 mM glycine, 0.6 mM ornithine, pH 6.8] in the presence and absence of 100μg/ml (0.6 mM) putrescine dihydrochloride and 0.15 μg/ml (0.6 μM) K_2_TeO_3_ [15] at 37°C with shaking at 120 rpm. Cell growth was monitored by measuring absorbance at 540 nm. Cell viability was determined by counting colony numbers grown on a Luria-Bertani (LB)-containing 1.5% agar plate at 37°C for 24 h.

### Plasmids

Total chromosomal DNA from *E*. *coli* W3110 was prepared according to the method of Wilson et al. [23]. To make pMW-lacSoxR, PCR was performed using total chromosomal DNA as template and 5’-CGACTGGATCCATGTTAAGCGGCTGGTCAA-3’ (P1) and 5’-ACCACGAATTCGAATGAGGTGTGTTGACGT-3’ (P2) as primers. The BamHI and EcoRI fragment containing *soxR* gene was inserted into the same restriction site of a low copy number vector pMW119 (Nippon Gene). Site-directed mutagenesis by overlap extension using PCR [24] was performed to prepare pMW-lacSoxR(SD). To make pMW-lacSoxR(SD), the first PCR was performed using P1 and P2(SD) (5’-CGACCTCGGAGAAGTTAACTTGAGGAATTA-3’) and P1(SD) (5’-TAATTCCTCAAGTTAACTTCTCCGAGGTCG-3’) and P2 as primers, and pMW-lacSoxR as a template. The second PCR was performed using the first PCR products as templates and P1 and P2 as primers. After cutting with BamHI and EcoRI, the PCR fragment was inserted into the same restriction site of pMW119.

To make the *soxR-lacZ* fusion gene, PCR was performed using total chromosomal DNA as template and 5’-GGCATAACCCGGGTCCATTGCGATATCAAA-3’ (P3) and 5’-CTCCCGGGGATACTGGTAATCAACCCTTTA-3’ (P4) as primers. The amplified *soxR* gene (a 326-nucleotide 5’-upstream region and a 120-nucleotide open reading frame) was digested with XmaI and inserted into the same restriction site of pMC1871 [25] to make the pMC*soxR-lacZ* fusion plasmid. For construction of pMW*soxR-lacZ*, the SalI fragment containing the *soxR-lacZ* gene of pMC*soxR-lacZ* was inserted into the same restriction site of pMW119. Plasmid pMW*soxR(SD)-lacZ* was prepared as described above with site-directed mutagenesis by overlap extension using PCR [24]. The first PCR was performed using P3 and P2(SD) and P1(SD) and P4 as primers.

Other plasmids [pMW-lacEmrR, pMW-lacEmrR(SD), pMW*emrR-lacZ*, pMW*emrR(SD)-lacZ*, pMW-lacGshA, pMW-lacGshA(ATG), pMW*gshA-lacZ*, pMW*gshA(ATG)-lacZ*] were constructed as described above. A list of oligonucleotide primers used was shown in S1 Table. The nucleotide sequence of the plasmids was confirmed by the 3130 Genetic Analyzer (Applied Biosystems).

### Dot Blot Analysis

*E*. *coli* MA261 cells were cultured at A_540_ = 0.05 in the presence and absence of putrescine and K_2_TeO_3_ as described above, and harvested at 24 h. Total RNA was prepared from these cells by the method of Emory and Belasco [26]. Dot blot analysis was performed according to the standard method [27] using the ECL direct nucleic acid labeling and detection systems (GE Healthcare Bio-Sciences). Oligonucleotide primers used for amplification of probes were shown in S1 Table. Chemical luminescence was detected by a LAS-3000 luminescent image analyzer (Fuji Film).

### Western Blot Analysis

Western blot analysis was performed by the method of Nielsen et al. [28], using ECL Western blotting reagents (GE Healthcare Bio-Sciences). Antibodies against SoxR, EmrR and GshA were prepared by injecting 1 mg each of SoxR, EmrR and GshA with Freund’s complete adjuvant to a rabbit [29]. Antibody against RpoS was prepared as described previously [30]. Antibody against β-galactosidase was obtained from Sigma-Aldrich. The level of protein on the blot was quantified with a LAS-3000 luminescent image analyzer (Fuji Film).

### Measurement of Polyamines, GSH and Carbonylated Proteins in Whole Cells

Polyamines were determined by high pressure liquid chromatography as described previously [31]. GSH was extracted from cells with 5% trichloroacetic acid (TCA), and measured using total glutathione assay kit (Northwest Life Science Specialties LLC, USA) according to the accompanying manual. Carbonylated proteins were measured using Western blot kit (Shima Laboratories Co., Japan) consisting of 2, 4-dinitrophenyl hydrazine and its antibody. Protein content was determined by the method of Bradford [32].

## Results

### Polyamine Stimulation of Cell Growth, Cell Viability and GSH Synthesis in the Presence of K_2_TeO_3_

**Fig 1:**
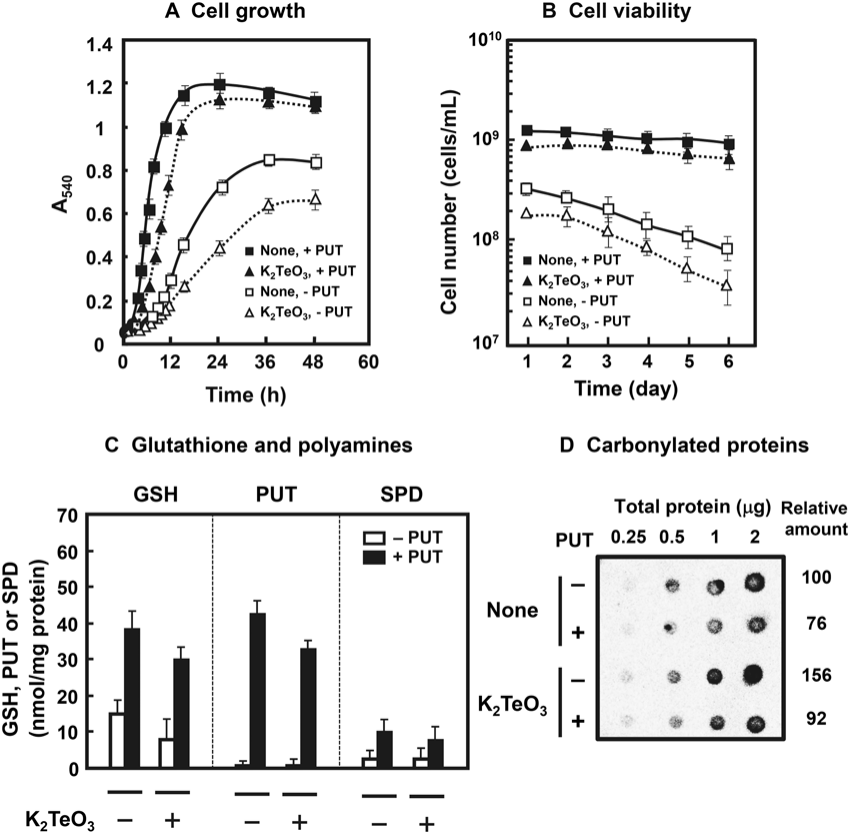
Effects of polyamines on cell growth and viability (A, B), the level of GSH and polyamines (C) and carbonylated proteins (D). These were measured as described in Materials and Methods. (A, B, and C) Values are mean ± SE of triplicate determinations. (C and D) Those were measured using cells cultured for 24 h. (D) The level of carbonylated proteins was measured using various amounts of proteins shown in the figure.

The effects of polyamines on cell growth and the level of GSH in cells were examined in the presence of 0.15 μg/ml K_2_TeO_3_, an inducer of oxidative stress [16], using a polyamine requiring mutant MA261. In this strain, putrescine is taken up into cells and spermidine can be synthesized from putrescine [13]. As shown in Fig 1A–1C, polyamines enhanced cell growth, cell viability and the level of GSH in cells in the absence or presence of K_2_TeO_3_. The degree of polyamine stimulation was greater in the presence of K_2_TeO_3_ than in the absence of K_2_TeO_3_. Furthermore, the level of carbonylated proteins, a marker of oxidative stress [33], was reduced in the presence of polyamines (Fig 1D). The polyamine effect on the decrease in carbonylated proteins was also greater in the presence of K_2_TeO_3_. The results suggest that the expression of some genes encoded by polyamine modulon is involved in these phenomena.

### Identification of Genes for SoxR, EmrR and GshA as Members of Polyamine Modulon under Oxidative Stress Conditions

**Fig 2:**
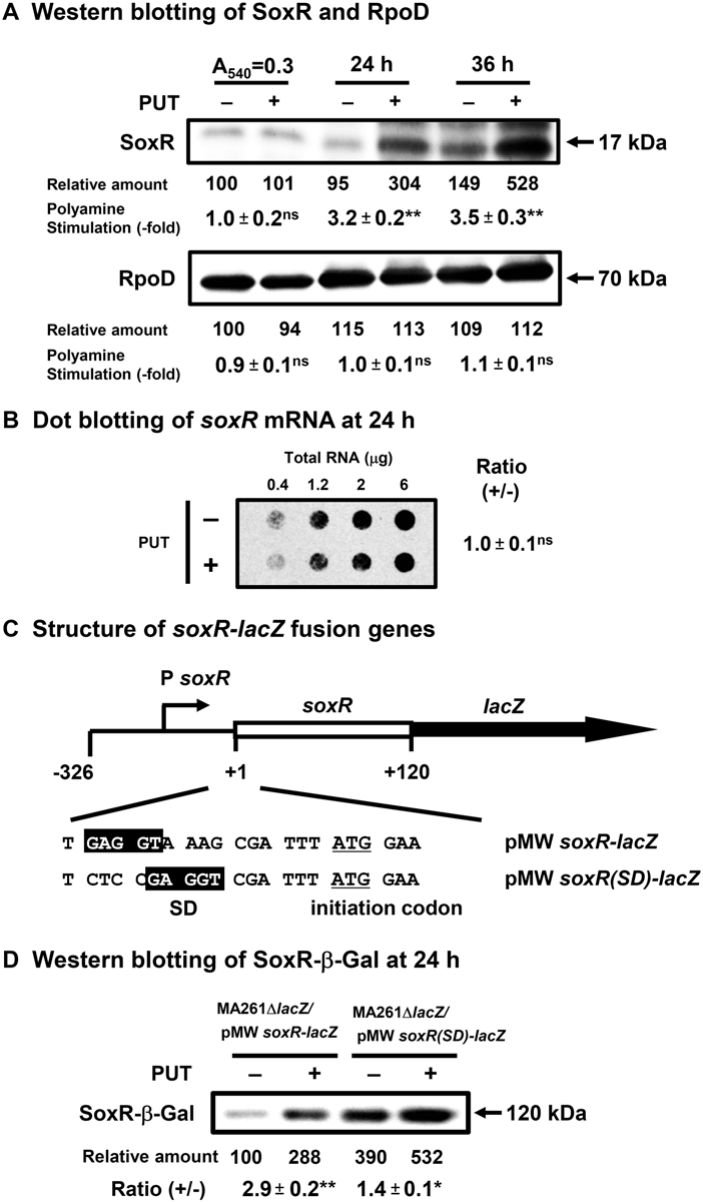
Levels of SoxR in *E*. *coli* MA261 cultured in the presence of 0.6 μM K_2_TeO_3_ with or without 100 μg/ml (0.6 mM) putrescine. (A) Cells were harvested at A_**540**_ = 0.3, or at 24 and 36 h after the onset of cell culture. Western blot analysis was performed using 20 μg protein. (B) Dot blot analysis of *soxR* mRNA in cells harvested at 24 h was performed using various amounts of RNA shown in the figure. (C) Structure of initiation regions of wild type and mutated *soxR-lacZ* genes was shown. The 40 amino terminal amino acid residues of SoxR are included in the fusion protein. (D) Western blot analysis of proteins in cells harvested at 24 h was performed using 5 μg protein and antibody against β-galactosidase. Values are means ± S. E. of triplicate determinations. Student’s *t* test was performed for the value obtained in the presence of putrescine versus in the absence of putrescine. ns, *p* ≥ 0.05; * *p* < 0.05; ** *p* <0.01.

As shown in Fig 2A and 2B, the synthesis of SoxR, a transcription factor for expression of the superoxide response regulon [17, 18], was not observed at A_540_ = 0.3 and was stimulated by polyamines at the level of translation about 3.2- and 3.5-fold in the presence of K_2_TeO_3_ at 24 h and 36 h, respectively, after the onset of cell growth. The results indicate that SoxR is preferentially synthesized at the stationary phase. The degree of polyamine stimulation of SoxR at 24 h was only 2.0-fold in the absence of K_2_TeO_3_ (data not shown). The synthesis of RpoD protein, σ^70^ transcription factor, was examined as a control, and it was not stimulated by polyamines (Fig 2A). The mechanism of polyamine stimulation of SoxR synthesis was studied using a *soxR-lacZ* fusion gene and *E*. *coli* MA261 *lacZ*::*Em*, in which there is no expression of β-galactosidase (β-Gal). The Shine-Dalgarno (SD) sequence of *soxR* mRNA was 10 nucleotides distant from the initiation codon AUG (Fig 2C), so the protein synthetic activity was measured after replacement of this unusual SD sequence with a SD sequence at the normal position 6 nucleotides from the initiation codon AUG. As shown in Fig 2D, after replacement of the unusual position of SD sequence with one at the normal position, the degree of polyamine stimulation of SoxR-β-Gal synthesis decreased from 2.9-fold to 1.4-fold, although the synthesis of SoxR-β-Gal protein in the absence of polyamines increased 3.9-fold. The results indicate that the *soxR* gene is a member of the polyamine modulon under oxidative stress conditions.

**Fig 3:**
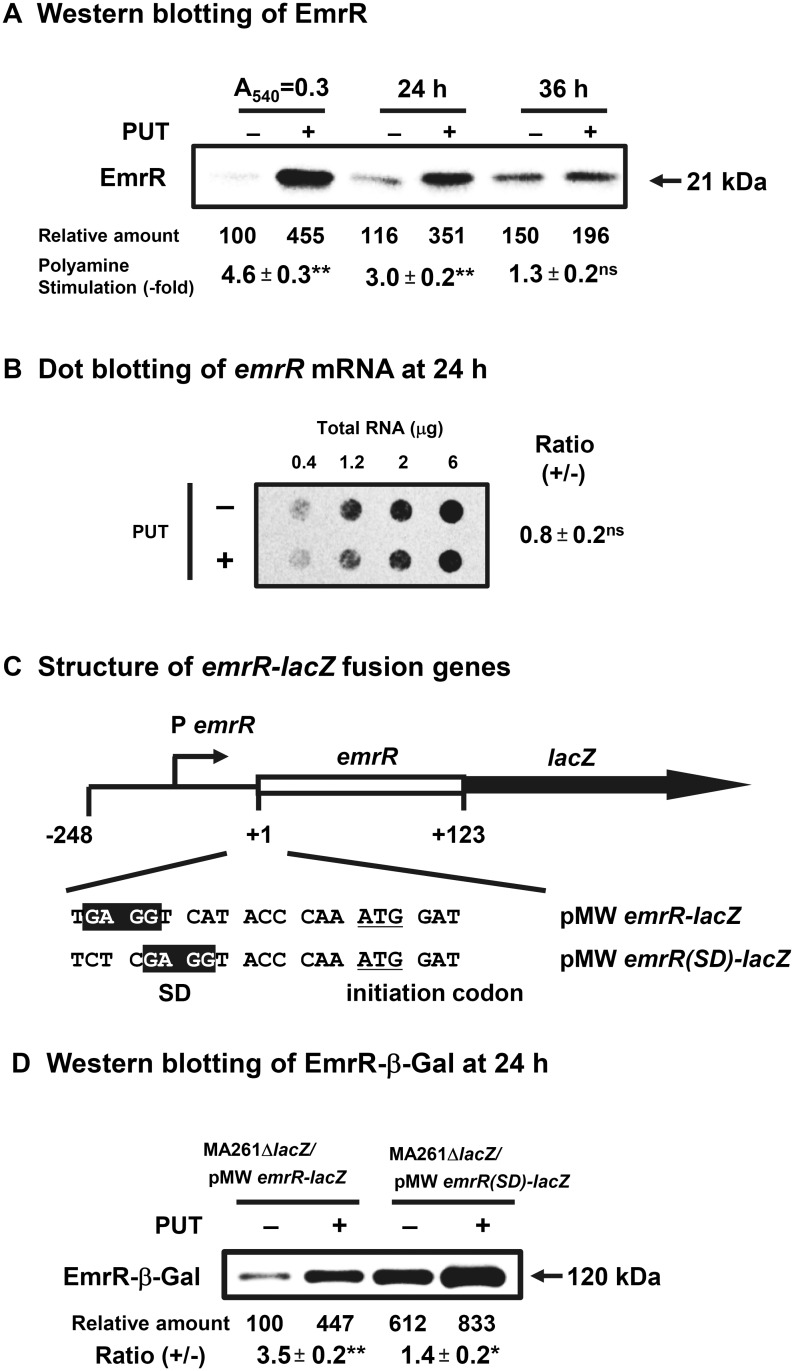
Level of EmrR in *E*. *coli* MA261 cultured in the presence of 0.6 μM K_2_TeO_3_ with or without 100 μg/ml (0.6 mM) putrescine. Experiments were performed as described in the legends of Fig 2. (C) The 41 amino terminal amino acid residues of EmrR are included in the fusion protein.

The synthesis of EmrR, a negative transcriptional factor for expression of the genes for drug excretion proteins, EmrA and EmrB [19], was increased by polyamines by 4.6-fold at A_540_ = 0.3, 3.0-fold at 24 h, and 1.3-fold at 36 h after the onset of cell growth (Fig 3A). The degree of polyamine stimulation of EmrR synthesis at 24 h in the absence of K_2_TeO_3_ was 2.5-fold (data not shown). The results also indicate that EmrR is preferentially synthesized at the logarithmic phase. Synthesis of EmrR was stimulated at the level of translation (Fig 3B). The SD sequence of *emrR* mRNA was also 10 nucleotides distant from the initiation codon AUG (Fig 3C). When EmrR-β-Gal synthesis was measured after the replacement of this unusual SD sequence with a SD sequence at the normal position, the degree of polyamine stimulation decreased from 3.5-fold to 1.4-fold, although the synthesis of EmrR-β-Gal protein in the absence of polyamines increased 6.1-fold (Fig 3D). The results indicate that *emrR* gene is another member of the polyamine modulon under oxidative stress conditions.

**Fig 4:**
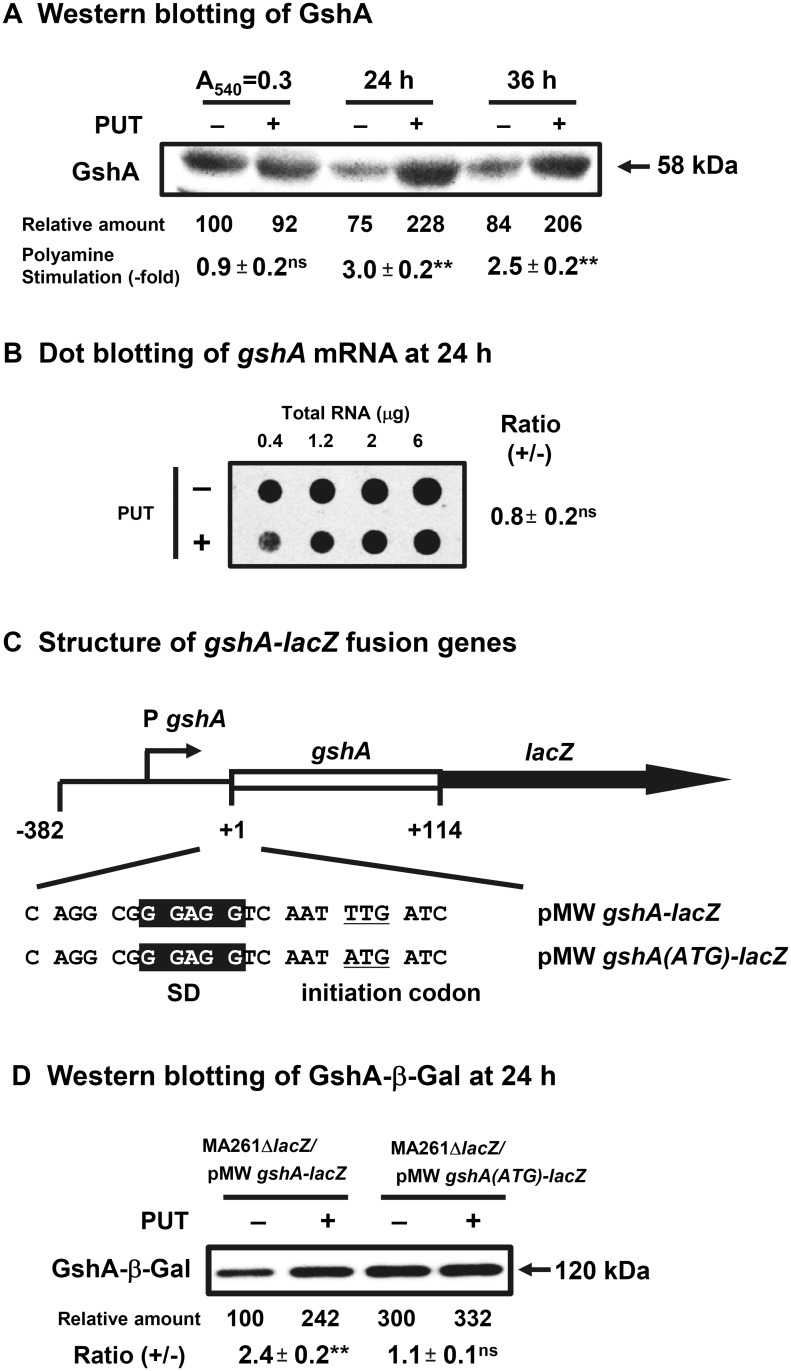
Level of GshA in *E*. *coli* MA261 cultured in the presence of 0.6 μM K_2_TeO_3_ with or without 100 μg /ml (0.6 mM) putrescine. Experiments were performed as described in the legends of Fig 2. (C) The 38 amino terminal amino acid residues of GshA are included in the fusion protein.

Reduced GSH is important for defense against oxidative stress [34]. It was found that the synthesis of GshA, γ-glutamylcysteine synthetase catalyzing the first step of glutathione synthesis [20], was enhanced 3.0-fold at the level of translation at 24 h after the onset of cell growth (Fig 4A and 4B). The results indicate that GshA is preferentially synthesized at the stationary phase. The degree of polyamine stimulation of GshA synthesis at 24 h slightly decreased to 2.0-fold in the absence of K_2_TeO_3_ (data not shown). The initiation codon of *gshA* mRNA was an inefficient codon UUG (Fig 4C), so the protein synthetic activity was measured after converting to an efficient initiation codon AUG. The synthesis of GshA-β-Gal protein from wild type mRNA was increased 2.4-fold by polyamines, but stimulation by polyamines was decreased to 1.1-fold after replacing the initiation codon UUG with AUG, although baseline synthetic activity, in the absence of polyamines, was greatly increased (3.0-fold). The results indicate that *gshA* gene is the third member of the polyamine modulon under oxidative stress conditions.

### Effects of SoxR, EmrR and GshA on Cell Growth and Viability

**Fig 5:**
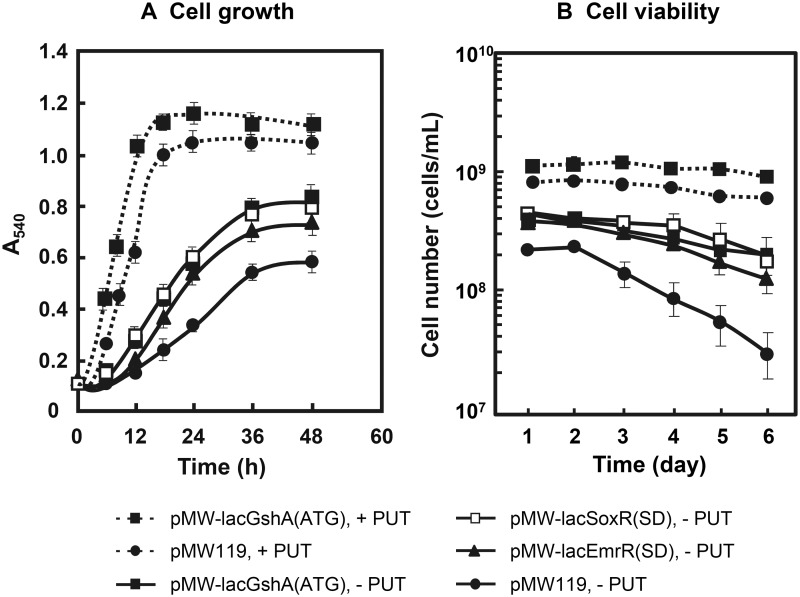
Recovery of cell growth (A) and viability (B) under polyamine deficiency by SoxR, EmrR and GshA. (A) *E*. *coli* MA261 carrying various plasmids shown in the figure were cultured for 2 days in the medium A with 0.5 mM isopropyl-β-thiogalactoside in the presence and absence of 100 μg/ml (0.6 mM) putrescine and presence of 0.6 μM K_**2**_TeO_**3**_. (B) Cell viability of the above cells was measured using 10 μl of bacterial cell culture every day during the culture for 6 days. Values are mean ± SE of triplicate determinations.

We next studied the effects of three genes on cell growth and viability of *E*. *coli* MA261 cultured in the presence and absence of polyamines and in the presence of K_2_TeO_3_. The genes used for these experiments were modified genes in which *soxR* and *emrR* genes were modified in the position of SD sequence [*soxR(SD)* and *emrR(SD)*] and *gshA* gene was modified in the initiation codon [*gshA(ATG)*]. As shown in Fig 5A and 5B, an increase in cell growth and viability was observed by transformation of these genes in the absence of putrescine. The degree of increase in cell growth and viability was in the order *gshA(ATG)* ≈ *soxR(SD)* > *emrR(SD)*. Effects of *gshA(ATG)* gene on cell growth and viability in the presence of putrescine were small compared with those in the absence of putrescine (Fig 5A and 5B). Similar results were obtained by *soxR(SD)* and *emrR (SD)* in the presence of putrescine (data not shown).

**Fig 6:**
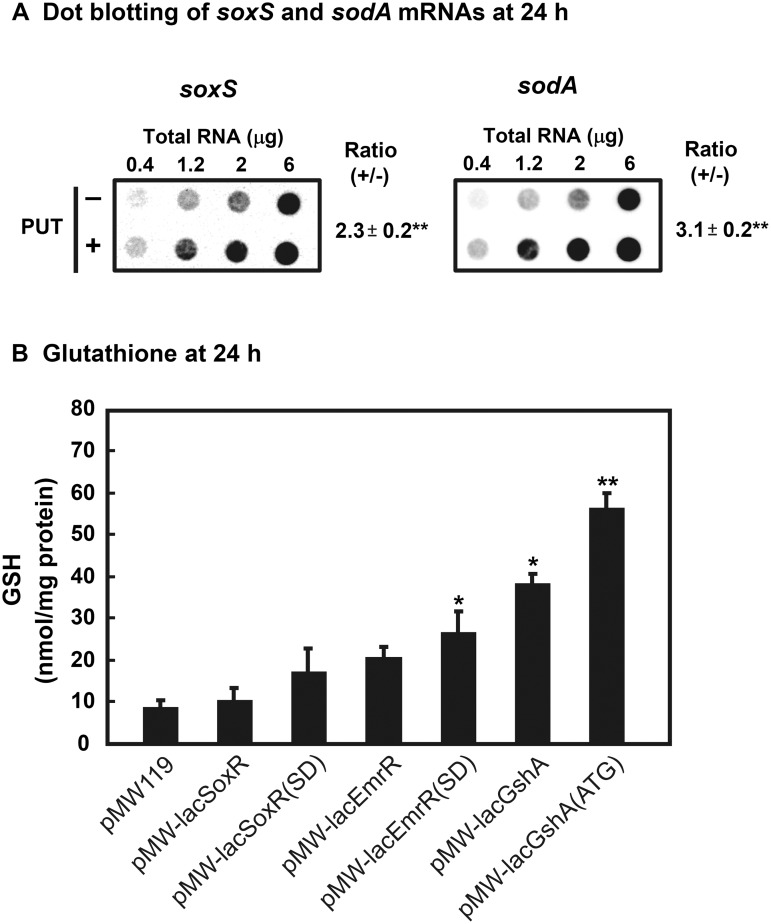
Levels of *soxS* and *sodA* mRNAs (A) and GSH (B). (A) *E*. *coli* MA261 cells were cultured for 24 h with or without 100 μg/ml (0.6 mM) putrescine and presence of 0.6 μM K_**2**_TeO_**3**_. Dot blot analysis of *soxS* and *sodA* mRNAs was performed after cells were cultured further for 30 min in the presence of 2 μM K_**2**_TeO_**3**_. (B) The level of GSH was measured using *E*. *coli* MA261 carrying various plasmids shown in the figure cultured for 24 h in the absence of putrescine with 0.6 μM K_**2**_TeO_**3**_. Values are mean ± SE of triplicate determinations. *, *p* < 0.05; **, *p* < 0.01.

The role of each gene on cell growth and viability was subsequently studied. SoxR is expected to increase the transcription of *soxS* gene encoding superoxide response regulon transcription activator and *sodA* gene encoding superoxide dismutase [35, 36]. As shown in Fig 6A, the levels of *soxS* and *sodA* mRNAs were increased 2.3- and 3.1-fold, respectively, in the presence of putrescine and K_2_TeO_3_. EmrR is expected to decrease the transcription of *emrAB* genes encoding multidrug resistance efflux complex [19]. Since it is suggested that EmrAB complex catalyzes efflux of cysteine [37], a substrate for the synthesis of GSH, increase in the level of GSH may be observed by overproduction of EmrR. As shown in Fig 6B, the level of GSH in cells was greater in cells transformed with pMW-lacEmrR(SD). Furthermore, the level of GSH in cells was much higher when pMW-lacGshA or pMW-lacGshA(ATG) was transformed (Fig 6B). Taken together, the results indicate that oxidative stress in *E*. *coli* is protected by polyamines, which stimulate the synthesis of two transcription factors SoxR and EmrR, and of GshA.

### Increase in the Degree of Polyamine Stimulation of RpoS Synthesis in the Presence of K_2_TeO_3_

**Fig 7:**
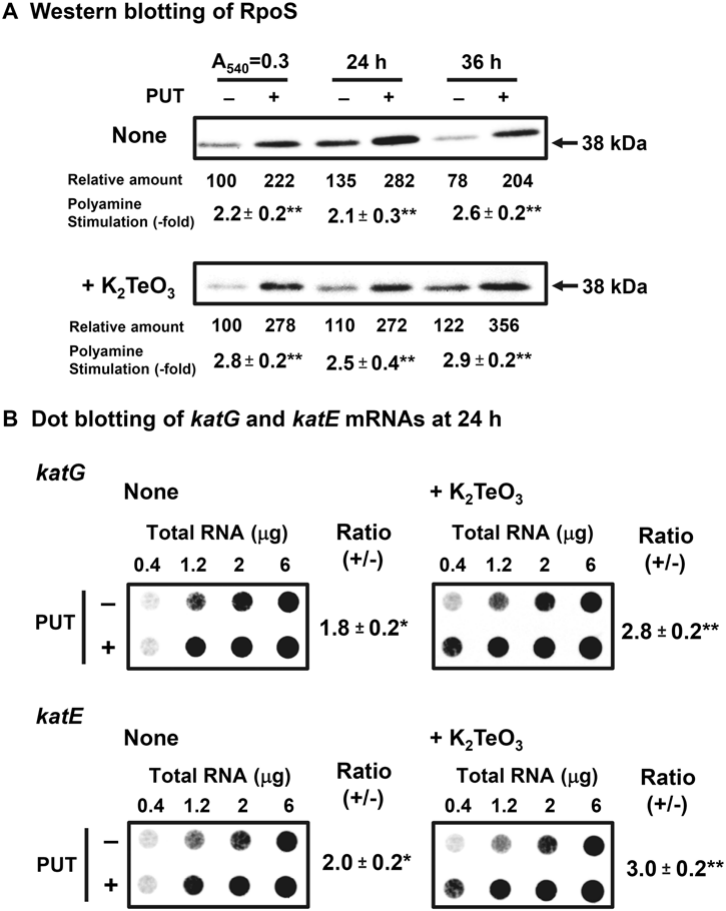
Levels of RpoS protein (A) and catalase mRNAs (B). *E*. *coli* MA261 cells were cultured for 24 h with or without 100 μg/ml (0.6 mM) putrescine and 0.6 μM K_**2**_TeO_**3**_. (A) The level of RpoS protein was measured by Western blotting using 20 μg protein. (B) Dot blotting for measurement of *katG* and *katE* mRNAs was performed using various amounts of RNA shown in the figure. Values are mean ± SE of triplicate determinations. *, *p* < 0.05; **, *p* < 0.01.

We have previously reported that synthesis of RpoS is enhanced by polyamines at the level of translation [38]. Thus, it was tested whether the degree of polyamine stimulation of RpoS synthesis is enhanced by K_2_TeO_3_. As shown in Fig 7A, the degree of polyamine stimulation of RpoS synthesis increased in the presence of K_2_TeO_3_. It has been also reported that catalases HP I (KatG) and HP II (KatE) are strongly expressed in stationary phase, as they are induced by RpoS system [39]. It was confirmed that the transcription of *katG* and *katE* genes was enhanced by polyamines especially in the presence of K_2_TeO_3_ (Fig 7B).

## Discussion

**Fig 8:**
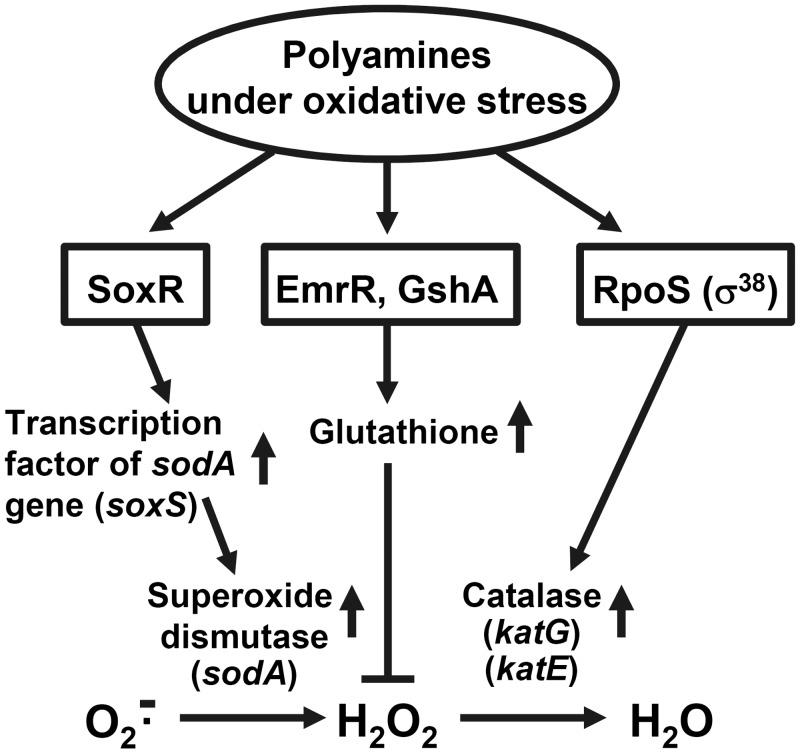
Role of SoxR, EmrR, GshA and RpoS (σ^38^) under oxidative stress. The figure summarizes how these proteins are involved in the decrease in cell toxicity of reactive oxygen species.

In this study, we looked for members of the polyamine modulon involved in oxidative stress. It was found that the synthesis of SoxR, EmrR and GshA was stimulated by polyamines at the level of translation, indicating that genes encoding *soxR*, *emrR* and *gshA* are members of polyamine modulon. The physiological significance of SoxR, EmrR, GshA and RpoS under oxidative stress is summarized in Fig 8. In case of *E*. *coli*, oxidative stress is mainly caused by superoxide (O_2_•^-^) and hydrogen peroxide (H_2_O_2_) [40–42]. Superoxide is detoxified by superoxide dismutases (SODs), and hydrogen peroxide is detoxified by GSH peroxidase [15], catalases (KatG and KatE) and hydroperoxide reductase (Ahp) [42]. Since SoxR is a transcription factor for expression of the genes for SODs, it stimulated the transcription of *sodA* gene (Fig 6). Furthermore, the level of GSH was increased through polyamine stimulation of the synthesis of EmrR and GshA (Fig 6). GshA directly increases GSH synthesis. Stimulation of EmrR synthesis probably causes the inhibition of the efflux of many substances with small molecular weight, which include GSH. Thus, hydrogen peroxide is detoxified effectively by GSH peroxidase through the increase of GSH. It was also shown that transcription of genes for *katG* and *katE* is enhanced through polyamine stimulation of RpoS synthesis (Fig 7). These results strongly suggest that polyamines are involved in easing of oxidative stress through stimulation of these proteins.

Oxidative stress is also weakened by OxyR, a positive regulator of hydrogen peroxide-inducible genes [43], through stimulation of the synthesis of AhpC and AhpF [42]. However, synthesis of OxyR was not influenced by polyamines (data not shown). Thus, this pathway is probably not involved in polyamine enhancement of cell growth and cell viability in the presence of K_2_TeO_3_.

Polyamine stimulation of SoxR and GshA synthesis was maximal at the stationary phase, but that of EmrR synthesis was maximal at the logarithmic phase. This may be reasonable, because cells started to save useful compounds necessary for survival under stress conditions, and then cells synthesize superoxide dismutase, glutathione synthase and catalase in the presence of polyamines for detoxification against superoxide and hydrogen peroxide. Thus, polyamines contribute to cell survival under oxidative stress conditions through stimulation of synthesis of SoxR, EmrR, GshA and RpoS.

We have thus far reported that synthesis of 17 proteins is stimulated by polyamines at the level of translation [14]. Synthesis of these proteins was stimulated by polyamines even in the presence of 0.6 μM K_2_TeO_3_ (data not shown). Thus, these proteins are also involved in the increase in cell growth and viability under oxidative stress. In addition, it has been reported that spermidine and spermine themselves function for protection against oxidative damage caused by hydrogen peroxide in *E*. *coli* and mammalian cells [44, 45].

## Supporting Information

S1 Table List of primers used for construction of various plasmids and probes for PCR. (PDF)
