# Supplementary material for: Three Members of Polyamine Modulon under Oxidative Stress Conditions: Two Transcription Factors (SoxR and EmrR) and a Glutathione Synthetic Enzyme (GshA)
Source: PLoS One. 2015 Apr 21;10(4):e0124883. doi: 10.1371/journal.pone.0124883 (PMC4405209; doi:10.1371/journal.pone.0124883)
Supplement: S1 Table — (PDF) [file pone.0124883.s001.pdf]

S1 Table List of primers used for construction of various plasmids and probes for PCR

No.	Primer used	Nucleotide sequence
Various plasmids		
P1	5'-soxR(BamHI)	5'- CGACTGGATCCATGTTAAGCGGCTGGTCAA -3'
P2	3'-soxR(EcoRI)	5'- ACCACGAATTCGAATGAGGTGTGTTGACGT -3'
P3	5'-soxR(SD)	5'- TAATTCCTCAAGTTAACTTCTCCGAGGTCG -3'
P4	3'-soxR(SD)	5'- CGACCTCGGAGAAGTTAACTTGAGGAATTA -3'
P5	5'-soxR(XmaI)	5'- GGCATAACCCGGGTCCATTGCGATATCAAA -3'
P6	3'-soxR(XmaI)	5'- CTCCCGGGGATACTGGTAATCAACCCTTTA-3'
P7	5'-emrR(BamHI)	5'- TGATGCAGGATCCACGCCGTTTCGTGCCCA -3'
P8	3'-emrR(EcoRI)	5'- TCTCGTGAATTCCCTGAGCCTTCAGCATTT -3'
P9	5'-emrR(SD)	5'- GGCTGAAATTAATGAGGAGGTACCCAAATG -3'
P10	3'-emrR(SD)	5'- CGAACTATCCATTTGGGTACCTCCTCATTA -3'
P11	5'-emrR(XmaI)	5'- TCTACCCGGGCAGAAGTGATGCACGATACA -3'
P12	3'-emrR(XmaI)	5'- TTAATCCCCGGGGCCTTCAGCATTTTATTG -3'
P13	5'-gshA(BamHI)	5'- TAGGCCTGGATCCATGGTCACCATTACAGT -3'
P14	3'-gshA(KpnI)	5'- CTAACAACGGCATTTAGGTACCTCCGGTAA -3'
P15	5'-gshA(ATG)	5'- GATTTTGACAGGCGGGAGGTCAATATGATC -3'
P16	3'-gshA(ATG)	5'- TGTGATACGTCCGGGATCATATTGACCTCC -3'
P17	5'-gshA(XmaI)	5'- GATCAGTCATCCCCGGGTTTTAGTTTTAAC -3'
P18	3'-gshA(XmaI)	5'- ATTGTTGCCCGGGTGCCATCAGCATTAACA -3'
Probes for PCR		
P19	5'-soxR(probe)	5'- CGACTGGATCCATGTTAAGCGGCTGGTCAA -3
P20	3'-soxR(probe)	5'- CTGCGCCAAAGGCAGCCACAACCAATACAT -3'
P21	5'-emrR(probe)	5'- TGATGCAGGATCCACGCCGTTTCGTGCCCA -3'
P22	3'-emrR(probe)	5'- ATCGCTTCGAGAACCACACCGTCTTGTTCC -3'
P23	5'-gshA(probe)	5'- TAGGCCTGGATCCATGGTCACCATTACAGT -3'
P24	3'-gshA(probe)	5'- AGATATCACCGCACTTCGCTTGCCAGAATG -3'
P25	5'-soxS(probe)	5'-CCCCAACAGATGAATTAACGAACTGAACAC-3'
P26	3'-soxS(probe)	5'-TAAAAACGATCGCTGAAGGCGTCGAAACTG-3'
P27	5'-sodA(probe)	5'- CTGGAGATGAATATGAGCTATACCCTGCCA -3'
P28	3'-sodA(probe)	5'- CCTCATTGCAGCAGGCGGCAAATGATTATT -3'
P29	5'-katE(probe)	5'- ATCAGCCGCTCACGGTTATTTCCAGCCATA -3'
P30	3'-katE(probe)	5'- AATCGCCTGCTTCAATGGCTTCCCACAACT -3'
P31	5'-katG(probe)	5'- TCGTTCTAACCCACTGGGTGAGGACTTTGA -3'
P32	3'-katG(probe)	5'- GTGCTCGCCCAACCTAAACCTTGTTCTTCA -3'
